# Supplementary material for: The Warburg Effect and lactate signaling augment Fgf-MAPK to promote sensory-neural development in the otic vesicle
Source: eLife. 2020 Apr 27;9:e56301. doi: 10.7554/eLife.56301 (PMC7253172; doi:10.7554/eLife.56301)
Supplement: Source data 1. [file elife-56301-data1.docx]

| Figure 1B | | WT | | | | s*agd1* | | | | |
| --- | --- | --- | --- | --- | --- | --- | --- | --- | --- | --- |
| Spec. | Number of *isl2b:Gfp*+ SAG cells - Anterior | 24h 30h 36h 48h | | | | 24h 30h 36h 48h | | | | |
| 1  2  3  4 |  | 45  49  30 | 95  85  61 | 79  84 | 93  110  83  114 | | 14  22  12 | 34  27  26 | 53  61 | 49  65 |
| 1  2  3 | Number of *isl2b:Gfp*+ SAG cells - Posterior | 10  6  1 | 9  19  11 | 23  26 | 33  25 | | 2  0  0 | 9  13  14 | 23  18 | 25  35 |

| Figure 1G | | WT | | | | | | | s*agd1* | | | | | |
| --- | --- | --- | --- | --- | --- | --- | --- | --- | --- | --- | --- | --- | --- | --- |
| Spec. | Number of *ngn1*+ cells inside otic vesicle | 18h 20h 24h 28h 30h 36h | | | | | | | 18h 20h 24h 28h 30h 36h | | | | | |
| 1  2  3  4  5  6 |  | 42  46  41 | 47  68  56 | 46  63  52 | 28  27  20 | 5  25  20  14  24 | 5  12  7  11 | 37  24 | | 51  48  30  54 | 31  30  17  20 | 15  14 | 13  16  10  15  11  21 | 6  9  9 |

| Figure 1H | | WT | | | | | | s*agd1* | | | | | |
| --- | --- | --- | --- | --- | --- | --- | --- | --- | --- | --- | --- | --- | --- |
| Spec. | Number of *ngn1*+ cells outside otic vesicle | 18h 20h 24h 28h 30h 36h | | | | | | 18h 20h 24h 28h 30h 36h | | | | | |
| 1  2  3  4  5  6 |  | 18  32  32 | 35  39  13 | 57  82  40 | 105  101  84 | 64  53  92  89  70 | 72  76  53  69 | 17  11 | 8  6 | 14  12  43  34 | 72  60 | 46  58  50  51  39  32 | 46  25  57 |

| Figure 1Q | | WT | | | | | | s*agd1* | | | | | | |
| --- | --- | --- | --- | --- | --- | --- | --- | --- | --- | --- | --- | --- | --- | --- |
| Spec. | Number of *neurod*+ cells | 36h 48h _  Ant Post Total Ant Post Total | | | | | | 36h 48h _  Ant Post Total Ant Post Total | | | | | | |
| 1  2  3  4 |  | 165  194  164  175 | 12  27  27  11 | 177  221  191  186 | 166  200  178 | 51  35  46 | 217  235  224 | | 83  67  80 | 14  12  14 | 97  79  94 | 150  131  134 | 42  53  35 | 192  184  169 |

| Figure 1-figure supplement 1I | | WT | | | s*agd1* | | | |
| --- | --- | --- | --- | --- | --- | --- | --- | --- |
| Spec. | Number of Isl1+ SAG cells-Anterior | 30h 36h 48h | | | 30h 36h 48h | | | |
| 1  2  3  4  5  6  7  8  9  10  11  12  13  14  15  16 |  | 35  32  36  41  34  34  35  38  35  34  35  35  34  35  35 | 47  40  39  39  42  42  37  45  41  39  38  44  45  43  42  44 | 57  56  60  59  55  61  54  51  46  46  47 | | 9  17  17  12  11  19  18  16  14  18  16  18  19 | 25  25  24  29  24 | 43  35  32  37  39  40 |
| 1  2  3  4  5  6  7  8  9  10  11  12  13  14  15  16 | Number of Isl1+ SAG cells-Posterior | 9  11  11  11  13  12  15  17  12  13  14  17  12  11  13 | 18  15  17  16  18  16  20  17  15  23  17  20  21  21  18  20 | 25  27  24  29  28  27  28  22  24  24  24  26 | | 8  8  8  8  9  8  10  8  8  10  8  10  8 | 15  16  17  14  13 | 22  25  23  20  24  25 |

| Figure 1-figure supplement 1J | | WT | | | | | s*agd1* | | | |
| --- | --- | --- | --- | --- | --- | --- | --- | --- | --- | --- |
| Spec. | Number of TUNEL+, Brn3c:Gfp+ SAG cells | 24h 30h 36h 48h | | | | | 24h 30h 36h 48h | | | |
| 1  2  3  4 |  | 8  15  8 | 15  11  9  4 | 2  0 | 0  0  0  0 | 0  6  1  0 | | 7  11  0  0 | 1  5  2  0 | 0  0 |

| Figure 2E | | WT | | | | | s*agd1* | | | |
| --- | --- | --- | --- | --- | --- | --- | --- | --- | --- | --- |
| Spec. | Number of *brn3c:Gfp*+ hair cells | 36h 48h _  Ant Post Ant Post | | | | | 36h 48h _  Ant Post Ant Post | | | |
| 1  2  3  4  5  6  7  8  9  10  11  12  13  14  15  16  17  18 |  | 10  9  11  10  11  9  11  9  10  9  10  9  10  10  11 | 8  8  8  8  8  8  8  8  8  8  8  8  7  7  8 | 16  17  16  16  16  16  16  17  16  17  17 | 18  18  18  17  19  17  17  18  17  20 | 7  8  8  7  9  8  8  8  8  8  7  7  8  9  7  8  7  7 | | 6  6  6  5  7  6  6  6  5  5  7  5  5  5  6  7  6  6 | 13  14  13  12  12  13  12  13  12  12  13  13  12 | 13  15  14  13  15  12  14  14  13  13  14  14  14 |

| Figure 3-figure supplement 3C | | *pgk1-alt* morpholino injections | | | | | |
| --- | --- | --- | --- | --- | --- | --- | --- |
| Spec. | Number of Isl1+ SAG cells 32 hpf | tbMO1 + sbMO +  Control tbMO1 tbMO2 tbMO2 sbMO tbMO1 | | | | | |
| 1  2  3  4  5  6  7  8  9  10  11  12  13  14  15 |  | 49  54  48  57  50  41  55  49  56  51  41  58 | 33  33  53  37  37  38  40  47  42  27  35  39  25  37  41 | 39  41  42  49  47  44  36  38  48  35  50  34  48  35  32 | 30  37  37  41  50  45  36  47  40  31  47  44  34  36  37 | 37  33  31  36  28  28  36  34  27  35  34  33 | 32  38  33  42  31  39  29  41  28  33  36  28 |

| Figure 4Q | | Heat shocked 39°C, 1 hour  *pgk1-* *sagd1-*;  WT *pgk1- sagd1-* x *sagd1- sagd1- hs:pgk1 hs:pgk1* | | | | | | |
| --- | --- | --- | --- | --- | --- | --- | --- | --- |
| Spec. | Number of Isl1+ SAG cells 30 hpf |  |  |  |  |  |  |  |
| 1  2  3  4  5  6  7  8  9  10  11  12  13  14  15  16  17  18  19  20  21  22  23  24  25  26  27 |  | 44  42  43  41  41  46  45  46 | 26  26  28  24  27  27  25 | 39  41  42  49  47  44  36  38  48  35  50  34  48  35  32 | 39  40  41  41  42  49  44  40  41  37  42  40  46  46  43  47  41  47  43  42  43  42  47  40  42  40  44 | 29  27  30  31  25  28 | 51  43  41  41  38  42  39  56  41  40  39  53  50  40 | 49  56  53  46  41  49  50  53  53  54  40  52  39  52 |

| Figure 4S | | WT | | | *pgk1* | | |
| --- | --- | --- | --- | --- | --- | --- | --- |
| Spec. | Number of Isl1+ SAG cells-Anterior | 36h 48h 120h | | | 36h 48h 120h | | |
| 1  2  3  4  5  6  7  8  9  10  11  12 |  | 41  39  38  40  41  41  38  41  40  41  40  39 | 61  60  59  61  60  59  61  63  60  60  60  60 | 101  103  106  106 | 34  33  32  30  31  32  32  31  31  32  31  31 | 48  47  49  48  47  47  48  47  47  48  47  49 | 70  75  74  77 |
| 1  2  3  4  5  6  7  8  9  10  11  12 | Number of Isl1+ SAG cells-Posterior | 15  15  14  16  16  15  16  15  15  15  14  15 | 26  26  25  26  24  25  26  26  24  25  25  24 | 48  49  55  46 | 11  11  12  13  12  12  14  13  11  12  12  11 | 21  20  19  20  19  21  21  19  20  20  20  22 | 47  40  36  42 |

| Figure 4T | | WT | | | *pgk1* | | |
| --- | --- | --- | --- | --- | --- | --- | --- |
| Spec. | Number of hair cells  -Anterior | 36h 48h 120h | | | 36h 48h 120h | | |
| 1  2  3  4  5  6  7  8  9  10  11  12  13  14  15  16  17  18  19  20  21  22 |  | 10  11  10  10  10  9  10  10  11  10  10  11  11  10  9  10 | 16  17  16  16  15  16  17  16  16  16  15  15  16  15 | 54  53  52  52  54  51  52  53 | 8  8  9  7  8  9  8  8  8  8  7  8  8  9  7  8  8  8  9  9  8  8 | 12  12  11  11  12  13  12  11  13  11  11  12  12  11  11 | 44  40  42  41  42  44  41 |
| 1  2  3  4  5  6  7  8  9  10  11  12  13  14  15  16 | Number of hair cells  -Posterior | 8  9  8  9  8  8  7  8  8  9  8  9  9  9  8  8 | 17  17  16  17  18  17  27  16  17  16  16  16 | 51  52  50  52  53  51  50  50  51 | 7  6  5  7  6  6  7  6  6  6  5  6  6  7  6  9 | 14  12  11  13  12  13  12  12  12  11  13  12  12  12 | 45  43  41  41  42  41  42  40 |

| Figure 4-figure supplement 3A-D | | Olfactory Facial Glossopharyngeal  epithelium ganglion ganglion. _  WT *pgk1-* WT *pgk1-* WT *pgk1-* | | | | | |
| --- | --- | --- | --- | --- | --- | --- | --- |
| Spec. | Pixel area of *neuod*  expression  domain |  |  |  |  |  |  |
| 1  2  3  4  5  6  7  8  9  10  11  12 |  | 18743  19317  19639  17927  19873  18719  17915 | 14055  12834  15240  13115  12939  14544  16409  14851 | 6635  6704  7665  6430  6879  7412  6893  7918  6552  7402 | 4308  4978  5387  5671  6711  6588  5790  5923  4677  3953 | 2626  2593  1905  1983  3111  2897  2745  2889  2491  2294 | 1233  1146  1529  1752  1344  1752  1344  1572  1130  1444  1671  1268 |

| Figure 4-figure supplement 3E-F | | Trigeminal Reticulospinal  ganglion neurons _  WT *pgk1-* WT *pgk1-* | | | |
| --- | --- | --- | --- | --- | --- |
| Spec. | Number of  Acetylated Tubulin+ cells |  |  |  |  |
| 1  2  3  4  5  6  7  8  9  10  11  12  13  14  15  16  17  18 |  | 16  19  17  14  16  18  20  15  16  12  16 | 6  7  8  6  7 | 15  15  16  17  16  15  21  19  13  17  19  20  15  18  14  20  20  21 | 7  9  5  6 |

| Figure 4-figure supplement 4A | | *pgk1-*  WT *hs:pgk1-alt* *pgk1- hs:pgk1-alt* | | | |
| --- | --- | --- | --- | --- | --- |
| Spec. | Number of Isl1+ SAG cells 32 hpf |  |  |  |  |
| 1  2  3  4  5  6  7  8  9  10  11  12  13  14  15  16 |  | 51  43  47  47  48  50  58  47  44  54  50  54  53  43  51  53 | 56  53  53  59  47  43  52  47  46  49  48  56  49 | 34  38  38  35  44  31  41  41  46  39  38  35 | 40  37  44  38  32  32  35  27  37 |

| Figure 4-figure supplement 4B | | WT *pgk1-alt*-sbMO  control  *hs:pgk1-alt pgk1-alt-*sbMO | | |
| --- | --- | --- | --- | --- |
| Spec. | Number of Isl1+ SAG cells 30 hpf |  |  |  |
| 1  2  3  4  5  6  7  8  9  10  11  12  13 |  | 45  47  44  44  43  44  43  46  43  41  46  47 | 44  46  47  48  48  46  46  44  45  49  44  48  47 | 31  29  32  33  34  34  31  30  36  34 |

| Figure 4-figure supplement 6A | | WT *pgk1-* *sagd1-*  control *plg*-tbMO *pgk1*- *plg*-tbMO *sagd1*- *plg*-tbMO | | | | | |
| --- | --- | --- | --- | --- | --- | --- | --- |
| Spec. | Number of Isl1+ SAG cells 30 hpf |  |  |  |  |  |  |
| 1  2  3  4  5  6  7  8 |  | 45  44  46  42  42  44  41  43 | 40  39  41  42  41  43  46 | 26  28  24  26  25  27 | 19  21  25  24  20  17 | 28  25  29  29  31  30 | 29  25  24  27  20  26 |

| Figure 4-figure supplement 6B | | WT *pgk1-*  control *plg-*sbMO *pgk1- plg-*sbMO | | | |
| --- | --- | --- | --- | --- | --- |
| Spec. | Number of Isl1+ SAG cells 30 hpf |  |  |  |  |
| 1  2  3  4  5  6  7  8  9  10 |  | 43  43  52  40  49  46  40  43  46  42 | 45  43  40  49  39  39  38  41  42 | 35  35  36  30  36  29 | 27  28  35  35  36  22  27 |

| Figure 5B | | 2DG 2DG 2DG 2DG 3PO 3PO 3PO DCAQ  250uM 250µM 250µM. 100µM 25µM 40µM 100µM. 100µM  WT t=0h t=0h t=0h t=14h t=0h t=0h t=14h t=14h  control WT *sagd1- sagd1- pgk1- pgk1-*  WT WT WT WT WT | | | | | | | | | | |
| --- | --- | --- | --- | --- | --- | --- | --- | --- | --- | --- | --- | --- |
| Spec. | Number of Isl1+ SAG cells 30hpf |  |  |  |  |  |  |  |  |  |  |  |
| 1  2  3  4  5  6  7  8  9  10  11  12  13  14  15  16  17  18  19  20  21  22  23  24  25  26  27  28  29  30  31  32 |  | 45  40  48  42  42  45  49  46  45  42  41  41  41  46  43  41  42  42  41  41  40  39  42  39 | 24  22  34  25  30  28  31  31  33  29  32  29  32  30  26  33  30  27 | 32  27  26  28  32  28  29  27 | 22  27  26  28  28  26  20  22  26  32  29  35  29  29  31  31  32  23  28  28  28  30  25  26  23  27  22  27  25  24  24  28 | 26  28  26  28  24  27  27  25 | 25  23  24  25  19  25  26  24  31  32  27  22  22  24  24  24  25  30  23  31  24  26  27  26  29  23 | 32  33  32  33  29  37  24  34  27 | 31  25  26  24  25  26  27  29  24  26  20  22  24  19 | 28  25  25  21  28  27  29  29  27  28  23 | 22  20  23  28  27  23  25  27  25  21  27  27  24  24  26 | 39  33  35  33  31  28  34  34  31  33  31  33  29  32  40  34  34  33  31  36 |

| Figure 5C | | Gal Gal Gal UK UK UK Lactate Lactate  25uM 50µM 100µM 20µM 50µM 100µM 6.7mM 6.7mM  WT t=14h t=14h t=14h t=14h t=14h t=14h pH6.2 pH6.2 t=14h t=14h  control WT WT WT WT WT WT WT *pgk1-* WT *pgk1-* | | | | | | | | | | |
| --- | --- | --- | --- | --- | --- | --- | --- | --- | --- | --- | --- | --- |
| Spec. | Number of Isl1+ SAG cells 30hpf |  |  |  |  |  |  |  |  |  |  |  |
| 1  2  3  4  5  6  7  8  9  10  11  12  13  14  15  16  17  18  19  20  21  22  23  24  25  26  27  28 |  | 44  40  45  42  41  41  39  44  42  42  41 | 36  29  33  43  40  39  38  29 | 32  31  24  25  34  33  29  34  22  25  23  28  27 | 29  23  29  26  24  24  29  23  27  29  22  29  23  26  29 | 28  31  31  31  34  32  29  32  36  23  24  29 | 34  26  33  24  25  22  24  28  28  28  32  26 | 23  21  26  26  24  22  28  29  24  27  23  24 | 47  46  42  42  48  46  43  39  44  46  41  38  41  47  41  47  48  41 | 29  27  26  27  29  19 | 52  53  48  48  59  60  60  52  48  47  60  52  48  47  60  57  51  52  52  52  48  60 | 47  43  55  53  45  40  43  51  41  51  49  47  41  49  41  49  44  53  48  53  44  42  55  53  50  48  42  49 |

| Figure 5D | | Lactate 2DG 2DG Gal Gal CHC CHC  6.7mM 25µM + 100µM + 100µM +  Lactate Lactate Lactate  WT t=14h  control WT WT WT WT WT WT WT | | | | | | | |
| --- | --- | --- | --- | --- | --- | --- | --- | --- | --- |
| Spec. | Number of *brn3c:Gfp*+ hair cells 36 hpf |  |  |  |  |  |  |  |  |
| 1  2  3  4  5  6  7  8  9  10  11  12  13  14  15  16  17  18 |  | 21  13  18  16  13  13  21  19  15  18  16  17  17  19  17  12  16  18 | 15  14  15  21  14  18  18  12  19  12  13  15  16  13  20 | 11  14  11  8  9  12  13  12  16  10 | 10  12  12  11  13  14  14  14  17  16 | 11  15  13  15  6  14  10  10 | 18  22  15  16  17  13  16  12  12  16  15  13  14 | 12  13  6  5  8  9  8  7  10  6  9  6 | 13  13  14  9  13  16  8  12  11  17  10  9  15  15  11 |

| Figure 6C | | Lactate ATP Lactate ATP  6.7mM 1mM 6.7mM 1mM  WT WT WT *pgk1-* *pgk1-* *pgk1-* | | | | | |
| --- | --- | --- | --- | --- | --- | --- | --- |
| Spec. | Number of *etv5b*+ cells 18 hpf |  |  |  |  |  |  |
| 1  2  3  4 |  | 106  115  94  88 | 97  110  102  126 | 126  117  136 | 52  62 | 115  125 | 60  72  67  53 |

| Figure 6D | | Lactate ATP Lactate ATP  6.7mM 1mM 6.7mM 1mM  WT WT WT *pgk1-* *pgk1-* *pgk1-* | | | | | |
| --- | --- | --- | --- | --- | --- | --- | --- |
| Spec. | Number of *ngn1*+ cells 24 hpf |  |  |  |  |  |  |
| 1  2  3  4  5  6 |  | 33  45  56  39  53  49 | 50  51  72  60  79  51 | 44  44  38  25 | 25  17  19  19  19  17 | 46  38  44  35 | 18  19 |

| Figure 6E | | Gal 2DG Azide U0126 CHC  100µM 25µM 25µM 30µM 100µM  WT t=14h t=0h t=14h t=14h t=14h  control WT WT WT WT WT | | | | | |
| --- | --- | --- | --- | --- | --- | --- | --- |
| Spec. | Number of *ngn1*+ cells 24 hpf |  |  |  |  |  |  |
| 1  2  3  4 |  | 44  46  47 | 34  32  24 | 20  17  34  32 | 30  42  46  33 | 16  15  17  20 | 23  22  31 |

| Figure 6-figure supplement 2A | | U0126 U0126 U0126 LY LY LY  WT 10µM 20µM 30µM 10µM 20µM 30µM  control WT WT WT WT WT WT | | | | | | |
| --- | --- | --- | --- | --- | --- | --- | --- | --- |
| Spec. | Number of Isl1+ SAG cells 30 hpf |  |  |  |  |  |  |  |
| 1  2  3  4  5  6  7  8  9  10  11  12 |  | 45  43  42  43 | 25  30  27  25  28  23  24 | 28  27  26  29  31  28 | 25  29  31 | 42  41  39  40  41  40  43 | 39  42  37  39  37  43  40  41  37  38  38  41 | 42  46  41  41  39  40 |

| Figure 6-figure supplement 2C | | Lactate Lactate  WT 6.7mM 6.7mM  control WT *hs:dnfgfr1 hs:dnfgfr1* | | | |
| --- | --- | --- | --- | --- | --- |
| Spec. | Number of Isl1+ SAG cells 30 hpf |  |  |  |  |
| 1  2  3  4  5  6  7  8  9  10  11  12  13  14  15  16 |  | 44  46  48  43  42  43  45  43  48  48  46  43 | 55  50  59  55  51  49  51  55  58  51  52  50 | 41  28  39  28  36  42  40  29  25  27  30  30  28  38  37  29 | 39  33  44  30  37  27  42  34  40  35  36 |

| Figure 6-figure supplement 2B | | Lactate  Lactate U0216 +  WT 6.7mM 20µM U0126  control WT WT WT | | | |
| --- | --- | --- | --- | --- | --- |
| Spec. | Number of Isl1+ SAG cells 32 hpf |  |  |  |  |
| 1  2  3  4  5  6  7  8  9  10  11  12 |  | 50  46  52  44  45  53  48  58  49  43  44 | 62  54  49  53  50  56  61  58  56  57 | 36  31  30  32  38  37  29  37  32  34  32 | 28  31  29  35  37  33  31  37  38  29  33  36 |
